# Soil biodiversity in natural forests potentially exhibits higher resistance than planted forests under global warming

**DOI:** 10.3389/fpls.2023.1135549

**Published:** 2023-04-28

**Authors:** Jianqing Wang, Xiuzhen Shi

**Affiliations:** ^1^ Key Laboratory for Humid Subtropical Eco-Geographical Processes of the Ministry of Education, Institute of Geography, Fujian Normal University, Fuzhou, China; ^2^ State Key Laboratory for Subtropical Mountain Ecology of the Ministry of Science and Technology and Fujian Province, School of Geographical Sciences, Fujian Normal University, Fuzhou, China

**Keywords:** climate change, forest management, global warming, soil biodiversity, soil microbes, subtropical forest, forest conservation

## 1 Introduction

The Intergovernmental Panel on Climate Change (IPCC) has reported that global surface temperature will continue to rise until the middle of the century, and global warming of 1.5°C and 2°C will be exceeded during the century (IPCC, 2021). It is widely accepted that global warming will cause a mass extinction in the coming years (Geisen et al., 2019; Turney et al., 2020; Wang et al., 2021), and biodiversity protection is increasingly important. Forests stand for one of the most crucial ecosystems on Earth and reserve a large proportion of the global biodiversity (Lladó et al., 2017). Unraveling the changes in biodiversity in forest ecosystems under global warming is a challenging field of research in theoretical ecology that remains under-explored in forest ecosystems.

Forest soil is an important living repository of biodiversity, with interactions between microorganisms, fauna, and plants providing multiple functioning and services to human health (Banerjee & van der Heijden, 2023). Soil biodiversity played a vital role in the forest ecosystem, owing to its inherent complexity (the traits, sizes, functions, and types of soil organisms), directly or indirectly underpinning multiple functioning and services, such as nutrient cycling, carbon sequestration, vegetation health, plant growth as well as soil stability (Delgado-Baquerizo et al., 2020b; Shi et al., 2021; Wang et al., 2022a; Wang et al., 2023b). Furthermore, soil biodiversity is increasingly acknowledged as beneficial to human well-being, as a consequence of reducing disease-causing soil microbes and also improving the quality and quantity of water, air, and food (Wall et al., 2015). Yet, forest soil biodiversity is frequently inadvertently altered by human-induced global warming.

Forests distributed in the tropical and subtropical regions are highly concentrated habitats of the Earth’s terrestrial biodiversity (Alroy, 2017). However, subtropical and tropical forests are assumed to be more vulnerable to global warming than temperate forests due to the relatively narrow upper thermal limits and temperature variation in subtropical and tropical regions (Sentinella et al., 2020). To combat global warming, many countries have committed to restoring forest areas (Bastin et al., 2019; Lewis et al., 2019). Almost half of the global forest area is set to become plantations of commercial trees (Lewis et al., 2019). However, several studies have pointed out that the adaptability of soil biodiversity in plantations to global warming might be lower than that of natural forests (Gibson et al., 2011; Lewis et al., 2019). Here, we provide several aspects of consideration that natural forests may exhibit higher soil biodiversity resistance than planted forests with respect to global warming, especially in subtropical forests.

## 2 Natural forests vs. planted forests

Natural forests play a pivotal role in conserving soil biodiversity and maintaining multiple ecosystem functions and services (Edwards et al., 2014). However, two-thirds of the land area has been set aside for reforestation worldwide (Lewis et al., 2019). Although plantations can contribute to mitigating some of the detrimental impacts of deforestation on soil biodiversity, the resulting soil quality and functions are declining compared to natural forests (Veldkamp et al., 2020). Indeed, our previous study found that long-term *Cunninghamia lanceolate* plantation greatly increased the abundance of plant parasite nematodes, consequently threatening soil and plant health in subtropical China (Zheng et al., 2022). Delgado-Baquerizo et al. (2020a) pointed out that the relative abundance of soil-borne fungal plant pathogens increased with warmer temperatures based on a global field survey and a nine-year field experiment. And as a consequence, the impacts of natural forests and planted forests on soil biodiversity raise serious concerns under global warming.

First of all, compared to scarce human disturbance, planted forest development is frequently subjected to regular harvesting and clearing of plantations (Poorter et al., 2016). Once trees were harvested, the land is cleared for massive planting of saplings, and chemical fertilizers and pesticides are normally applied to facilitate the rapid accumulation of standing tree biomass. These plantation management practices possibly lead to forest land degradation, further decreasing soil biodiversity and consequent ecosystem multiple functions in subtropical forests (Wang et al., 2022a). By contrast, the natural forest possibly increases soil biodiversity by protecting land from fire and human disturbances and thus improving multiple ecosystem functions and services in the subtropical forest (Shi et al., 2021).

**Figure 1:**
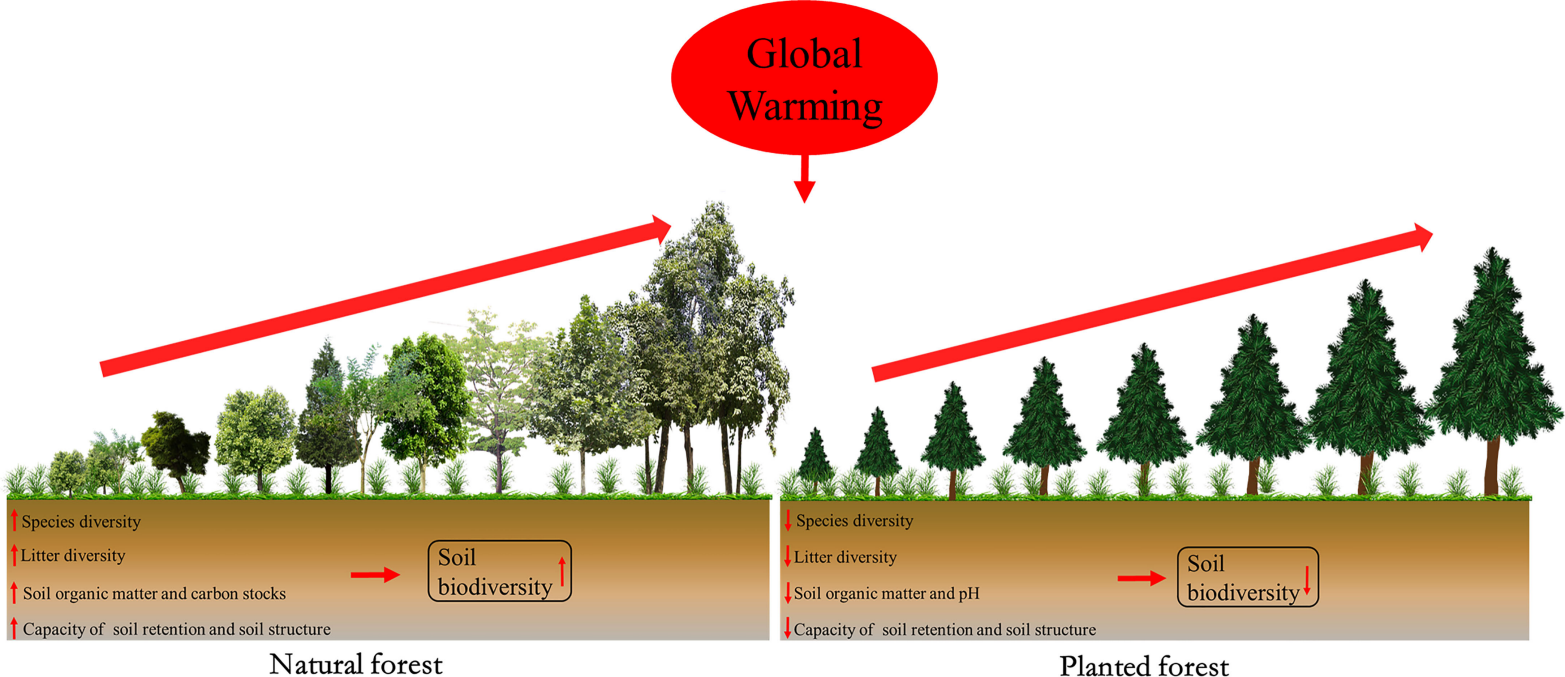
Conceptual graph of the changes in ecosystem processes and variables with forest restoration between natural and planted forests in the subtropical zone under global warming. The red upward arrow represents a positive effect; the red downward arrow indicates a negative effect.

Secondly, planted forests intrinsically comprise substantially lower tree diversity than native forests. However, to alleviate global warming, ongoing reforestation efforts accelerate the current loss of biodiversity (Carnus et al., 2006). We previously found that soil nematode abundance significantly increased with forest restoration and improved soil health status in natural forests rather than in planted forests in subtropical regions (Wang et al., 2022b; Zheng et al., 2022). This is mostly attributed to the fact that species-rich ecosystems can enhance soil microbial growth and biomass by providing a higher amount of plant-derived resources (e.g., litter inputs and root exudates) (**Figure 1**) (Prommer et al., 2020). It is commonly established that species-rich natural forests better support biodiversity than planted forests in the tropical zone (Gibson et al., 2011).

Thirdly, natural forests exhibit higher water use efficiency and soil organic matter storage compared to planted forests (Yu et al., 2019). The conversion of natural forests to planted forests caused substantial declines in soil organic carbon retention by around 60% (Zarafshar et al., 2020). Furthermore, carbon capture capacity in planted forests was less stable than in natural forests, particularly in the face of increasing global warming in the tropical zone (**Figure 1**) (Osuri et al., 2020). Ghosh et al. (2020) also highlighted that the quality and temperature sensitivity (Q_10_) of soil organic matter were higher in the natural ecosystem than in the managed ecosystems in North Eastern India, indicating protecting natural ecosystems is very important to mitigate climate warming. Amoo et al. (2021) revealed that planted forests consistently decreased multiple soil functions linked to soil carbon cycling and nutrient dynamics, and altered the functional profile and activity of soil microbial communities in South Africa. The planted forest can therefore have lasting detrimental impacts on soil biodiversity and health, in addition to posing a significant threat to terrestrial ecosystem functions in subtropical forests (Carnus et al., 2006; Wang et al., 2022a; Zheng et al., 2022). Therefore, we expect greater soil biodiversity in the species-rich natural forests than that in the species-poor plantations, particularly in subtropical forests.

Therefore, it is generally believed that natural forests can assist to relieve the negative impact of global warming by conserving biodiversity, as well as supporting multiple ecosystem functions and services (Alroy, 2017). In the future, forest natural restoration rather than planted forests would continue to be one of the most effective ways to combat global warming in subtropical forests.

## 3 Expectations in soil biodiversity study

Comparatively little is known about the responses of soil biodiversity, with most climate warming experiments concentrating on the aboveground ecosystems (Fierer et al., 2009). Soil organisms are an extremely diversified assemblage of organisms, which involves soil microorganisms (i.e., archaea, bacteria, and fungi), and soil fauna (i.e., protozoa, nematode) (Orgiazzi et al., 2016; Van Den Hoogen et al., 2019; Aslani et al., 2022). As important components of forest ecosystems, soil organisms undertake a wide range of ecosystem functions and services (Shi et al., 2021; Wang et al., 2023a), such as mediating biogeochemical cycling and ecosystem health maintenance, their responses to global warming are potentially important in subtropical forest ecosystems. However, the impacts of global warming on soil organisms and biodiversity are rather equivocal to date. Therefore, the authors encourage filling this knowledge gap from the perspective of the soil food web to thoroughly understand soil biodiversity, including vegetation resources, soil microorganisms, and soil fauna.

This is a research area receiving little attention in forest ecosystems but could potentially have a tremendous impact on combating global warming in the future. However, disentangling soil food webs and biodiversity in the forest soil may be a daunting task, because of the enormous phylogenetic variety in the soil (Kardol et al., 2016). Traditionally, soil web food studies have used identification procedures based strictly on morphological traits. This approach requires a high degree of taxonomic competence, which usually restricts the number of species that can be investigated concurrently (Oliverio et al., 2018). Furthermore, the procedures of morphological identification are time-consuming, and intensive efforts are required from taxonomists aiming to recognize biodiversity at lower taxonomic levels (Madden et al., 2016). In recent years, molecular analysis tools for soil food web and biodiversity are now widely used in soil ecology, and molecular methods have greatly improved our knowledge of soil biodiversity (Thomsen & Willerslev, 2015). Molecular ecological approaches, such as metagenome, ^13^C isotope identification, and network analysis, have been increasingly applied for the characterization of the soil food web and biodiversity (Shi et al., 2018; Chomel et al., 2022).

## 4 Conclusions

Overall, to mitigate the adverse impacts of global warming on soil biodiversity, we urge the restoration community, forestry specialists, and legislators to emphasize natural forest regeneration over various tree-planting approaches, thus maintaining soil biodiversity and improving multiple ecosystem functions. Additionally, we call for new theories and technologies to preserve soil biodiversity based on the soil food web to combat global warming in the future. This perspective will emphasize the necessity of strengthening natural regeneration rather than planted forests for maintaining soil biodiversity and ecosystem functioning.

## Author contributions

JW contributed ideas to the study and drafted the manuscript. XS contributed ideas to the study and improved the draft. All authors contributed to the article and approved the submitted version.
